# Maximizing the potential of psychology for the Israeli mental health reform

**DOI:** 10.1186/2045-4015-3-39

**Published:** 2014-11-27

**Authors:** Jonathan D Huppert

**Affiliations:** Department of Psychology, The Hebrew University of Jerusalem, Mt. Scopus, Jerusalem, 91905 Israel

## Abstract

The author comments on Nirel and Samuel’s article showing that psychologists in Israel reported practicing inconsistently with the likely demands of an upcoming Israel mental health reform. Some of the reasons for the differences in preparedness between psychologists and psychiatrists are considered. In addition, incorporating the knowledge from psychology to improve access, assessment, treatment and training in Israel is discussed as a way to help both the reform and psychologists advance treatments in Israel. Finally, a suggestion for 21^st^ century models of service delivery is suggested.

The article by Nirel and Samuel [1] describes the practices amongst psychologists and psychiatrists in Israel, in consideration of the upcoming mental health reform in Israel. Approximately one quarter of registered practicing psychologists and one half of psychiatrists responded to a survey regarding their practices and their views of the reform. Results indicated that psychiatrists work more in public institutions while psychologists work more privately. In comparison with psychologists, a greater proportion of the psychiatrists reported being required to provide a diagnosis for their patients. They also reported greater demands in documenting every step of the treatment process, and greater demands to document assessments of outcome (although in both groups, less than 50% reported being required to do this). In terms of practice, psychiatrists reported a more balanced range of short, medium, and long-term treatments, while psychologists reported that the large majority of their treatments were long term (more than a year). Psychiatrists also reported greater use of structured assessments and interviews, as well as more awareness and consideration of evidence-based practices in treatment planning. In many of these areas, very few psychologists (less than 20%) reported taking these issues into consideration in their practice.

Given the low level of preparedness for the reform described by psychologists, what are some steps that may facilitate success of mental health reform in Israel, and how can psychologists become more prepared so that they are appropriately integrated into the new model? I take the points raised in the article as a launching point to consider a number of essential issues related to the treatment of mental health in Israel and beyond: training, quality assurance, access, assessment, and treatment.

As Nirel and Samuel note, the goal of managed care is to cut costs while improving access. These two goals can appear to contradict one another. After all, the simplest way to cut costs in mental health care is to restrict or deny access, whereas providing unlimited treatment for anyone who requests it may be the simplest road to greater access. In a publicly-financed medical system, there is an attempt to balance these two objectives. In Israel prior to the reform, this has been done via provision of free behavioral health services via the Ministry of Health, with some percentage provided via the health management organizations (HMOs), and fee-for-service private practice (some of which may be covered by the HMOs, some of which is not). The reform transfers all non-private outpatient care to the HMOs, with intent to also capture a share of private practice. This raises many questions. The first is: What is the incentive of the HMOs to take on a service that will likely lose money, especially when they already often operate at a financial loss? And more to the current issue, what is the impact of cost-cutting efforts on access and quality of care for vulnerable populations?

Notably, one laudable goal of the reform is to create greater access to care for individuals who suffer from anxiety and depression. Indeed, the current model, which intermingles services for individuals with chronic mental illness (e.g., schizophrenia, bipolar disorder, personality disorders) with more acute disorders (anxiety and depressive disorders which, when treated with evidence-based methods, are less likely to follow a chronic course) creates a situation which is likely untenable, as those with the acute disorders may be less likely to seek treatment due to stigma, long waiting lists, and more^a^. In fact, one could interpret the data from Nirel and Samuel as supporting this contention (as most community mental health centers appear to see more of the former and other care providers see more of the latter). It may be the case that by integrating physical and behavioral healthcare as planned by the reform, more patients will be provided with “prescriptions” for treatment by their primary care physicians in ways that make them more likely to follow through (e.g., seeing a clinician in the same building as the primary care physician) and that are less stigmatizing.

Nurit and Samuel have demonstrated that psychiatrists and psychologists practice differently. One may ask: Why this is? One possibility is that psychiatric training emphasizes the biological model, medications, and the attempt to provide the fastest, most effective intervention. In contrast, the training of psychologists emphasizes the importance of providing the most thorough care, that, when done correctly, can both consider the whole person and also provide relief for many individuals, while also helping to prevent relapse. It is notable that most psychiatrists have been trained outside of Israel while most psychologists have been trained in Israel. Indeed, training received outside of Israel may be more likely to be in the context of managed care, and therefore may further accentuate the differences in philosophy between psychiatry and psychology as described above. In their training abroad, they may be trained to provide immediate relief in as short a time as possible (both in terms of the length of sessions and numbers of sessions). Recent changes in the managed care environment and psychiatric training programs influence the treatment toward medications, and there may also be bias due to drug company influence. At the same time, psychologists are likely to see patients who are either medication resistant or those who do not want to take medications. Despite the differences in their training, data do not support that psychiatrists or their unique interventions are any more effective than psychologists in their treatment outcomes (particularly for anxiety and depression).

According to the survey, psychologists in Israel engage primarily in private practice. Private practice, almost by definition, is just that: private. This means that the therapist, while accountable to the Psychology Law and standards of ethical practice, have no true overseer ensuring quality, follow-through with procedures, etc. This, on the one hand, means that any answers to questions about the requirements of their practice should inherently be different from the standards of services provided (more by psychiatrists) under the auspices of the government health ministry or the health management organizations (HMOs). The latter can make demands of standardized procedures, evaluations, and other service evaluations. Private practices have their own incentives, and there are few incentives to provide short term treatments, to diagnose, or to carry out standardized brief assessments and interviews. Thus, the nature of the practice of psychologists may simply be different, and it is not clear how private practices will be impacted by the reform.

In order to adapt to the demands of managed care, psychologists need not look beyond their own field. There are a number of important developments in the field of mental health that have been advanced by psychologists and that are related to improved efficiency and efficacy of treatments. In many countries, it is psychologists who are leading the efforts to improve access and quality of care. These developments include both assessment and treatment, and have implications for the training of future psychologists. First, psychologists have worked along with psychiatrists to develop the diagnostic systems and diagnostic interviews to determine diagnosis. Data support the contention that when such interviews are used, more comorbid disorders are identified, and both treating clinicians and their patients report that they want treatment to focus on the comorbidity (e.g., [2]). Given that only psychologists and psychiatrists in Israel have the right to diagnose psychiatric disorders, Nirel and Samuel’s data suggest that psychologists are opting out of an important role they can and should assume. In addition, there are many short, easily administered assessment instruments that can be used to determine severity and progress in treatment. Accumulating data suggest that repeated administration of these measures during treatment improves outcomes [3]. Some clinics have determined that these findings are so strong, that they feel it is unethical to study this further by withholding feedback systems from some patients [4]. Furthermore, there are multiple avenues spearheaded by psychologists, dedicated to improvements in access to care, saving costs, and decreasing the burden of depression and anxiety. This includes the Improving Access to Psychological Therapies (IAPT) program in the UK, which has been a success in doing both, meriting continued government funding [5]. This program, based on the idea of following evidence-based guidelines, is working on training over 6,000 clinicians with a goal of treating over 600,000 individuals who suffer from anxiety and depression. The outcome data support the overall efficacy of this effort.

However, as Kazdin and Blase note [6], individual and group psychotherapy efforts are, in many ways, 20^th^ century solutions. While there will always be individuals who want and need face-to-face treatment, not all who suffer from maladies such as anxiety and depression do. In fact, internet-based, therapist assisted self-help treatments have demonstrated great promise [7, 8]^b^. In Australia, such programs have been shown to yield benefits similar to face-to-face treatment with minimal therapist contact [9]. Furthermore, data suggest that populations that do not otherwise seek treatment may choose to seek such interventions, that those using internet based interventions are more representative of population demographics than those who attend public clinics, and that the two groups have similar levels of symptom severity [10]. In addition to internet-based interventions based on standard models of treatment, new, novel computerized treatments that are attempting to modify basic cognitive processes underlying anxiety and depression have begun to show promise (e.g., [11]). These 21^st^ century advances do not necessarily replace traditional face-to-face treatment, but instead may provide for new stepped care models where only those who fail or reject such interventions would move on to more intensive treatments. It is at later stages in stepped care that clinical acumen in building relationships with difficult to treat patients would continue to be essential. And it is likely that those not responding to first-line treatments will need longer, more intensive treatments to lead to greater improvements and maintenance of gains.

Overall, most advances in psychological services are developed on the basis of data collected from innovative paradigms throughout the world and are then readily disseminated. It is essential that data be collected now, before the institution of the reform, not only on the practices of psychologists and psychiatrists, but on who comes to the community mental health clinics and what their outcomes are, how to improve access, and how to increase reach to the many individuals who suffer from psychological distress. Only then can the reform be compared to this standard care to determine if it is a success. Data need to be used to determine if planned reforms actually lead to improved access, assessment, and treatment when compared with current practices.

In addition, there is a strong need to consider how to integrate psychology training within institutions carrying out the reform. Until now, training has been predominantly conducted by public mental health services, which are supposed to move under the auspices of HMOs. There is no plan on how training will continue.

Finally, the reform both gives rise to great potential for and demands of further research. First, more information is needed on accessibility and outcomes. Who are currently the main consumers of mental health services? What services do they receive? How effective are these services? More importantly, who are those individuals who are not receiving services, but could benefit greatly from them? What type of services would they be willing to access? Will integration between behavioral health and general health lead to greater service utilization? Will such services be effective (decrease symptoms, decrease work disability and unemployment)? Will following international guidelines improve outcomes? If not, what will? How can the core evidence-based practice principles of research, clinical experience, and patient values be best utilized to facilitate more effective and more efficient services to a greater number of those who need? How can the technologically savvy Israeli culture be captured in facilitating these services?

These questions, which can best be answered by multidisciplinary research teams, have great potential for bidirectional, cross-national learning. The reform can learn significantly from other mental health reforms (for example from the United Kingdom, Holland, and now in Norway). At the same time, there is opportunity to develop a modern, culturally adapted, technologically advanced, stepped care system that optimizes utilization of existing practitioners while providing more services to many more who need it. Adapting such a model on the national level could make the Israeli system not only effective, but one to be emulated in other countries. However, this can only happen if reliable data is collected and utilized throughout the process of the reform.

Psychologists, working along with other mental health specialists and public health experts, need to use their expertise in assessment, data collection, treatment development, and research design to ensure that an evidence-based reform will occur. Overall, there appears to be much potential in the reform: potential for good as well as potential for harm. In order for the former to be realized, it will take a great effort for all involved to work together to improve the system, for the good of those suffering.

## Endnotes

^a^While there is significant comorbidity between these artificially defined groups, there is a significant group of patients with anxiety and depressive disorders without other issues. It is not clear how many of these seek treatment in current public mental health clinics, despite the distress, interference, and economic burden that they face.

^b^Such interventions can be used for any evidence-based treatment. See Andersson et al. [12] for an example of internet based psychodynamic therapy.

## Author information

The author is an Associate Professor of Psychology at The Hebrew University of Jerusalem, and the former Director of Clinical Training of the clinical psychology program. He is an expert on the nature and treatment of anxiety and related disorders using psychosocial interventions such as cognitive-behavioral therapy.

## Commentary on

Nirel N, Samuel, H: Work practices and the provision of mental-health care on the verge of reform: a national survey of Israeli psychiatrists and psychologists. Israel Journal of Health Policy Research 2014, 3:25.

## References

[1] Nirel N, Samuel H (2014). Work practices and the provision of mental-health care on the verge of reform: a national survey of Israeli psychiatrists and psychologists. Isr J Health Policy Res.

[2] Zimmerman M, Chelminski I (2003). Clinician recognition of anxiety disorders in depressed outpatients. J Psychiatr Res.

[3] Lambert M (2007). Presidential address: What we have learned from a decade of research aimed at improving psychotherapy outcome in routine care. Psychother Res.

[4] Slade K, Lambert MJ, Harmon SC, Smart DW, Bailey R (2008). Improving psychotherapy outcome: The use of immediate electronic feedback and revised clinical support tools. Clin Psychol Psychother.

[5] Clark DM (2013). Developing and disseminating effective psychological treatments: Science, practice and economics. Can Psychol Psychol Can.

[6] Kazdin AE, Blase SL (2011). Rebooting psychotherapy research and practice to reduce the burden of mental illness. Perspect Psychol Sci.

[7] Andersson G, Cuijpers P, Carlbring P, Riper H, Hedman E (2014). Guided Internet‒based vs. face‒to‒face cognitive behavior therapy for psychiatric and somatic disorders: a systematic review and meta‒analysis. World Psychiatry.

[8] Andrews G, Cuijpers P, Craske MG, McEvoy P, Titov N (2010). Computer therapy for the anxiety and depressive disorders is effective, acceptable and practical health care: a meta-analysis. PLoS One.

[9] Andrews G, Williams AD: **Up-scaling clinician assisted internet cognitive behavioural therapy (iCBT) for depression: A model for dissemination into primary care.** *Clin Psychol Rev* in press 10.1016/j.cpr.2014.05.006 25043445

[10] Titov N, Andrews G, Kemp A, Robinson E (2010). Characteristics of adults with anxiety or depression treated at an internet clinic: comparison with a national survey and an outpatient clinic. PLoS One.

[11] Amir N, Beard C, Taylor CT, Klumpp H, Elias J, Burns M, Chen X (2009). Attention training in individuals with generalized social phobia: A randomized controlled trial. J Consult Clin Psychol.

[12] Andersson G, Paxling B, Roch-Norlund P, Östman G, Norgren A, Almlöv J, Silverberg F (2012). Internet-based psychodynamic versus cognitive behavioral guided self-help for generalized anxiety disorder: a randomized controlled trial. Psychother Psychosom.

